# Robot-assisted modified bilateral dismembered V-shaped flap pyeloplasty for ureteropelvic junction obstruction in horseshoe kidney using KangDuo-Surgical-Robot-01 system

**DOI:** 10.1590/S1677-5538.IBJU.2022.0525

**Published:** 2022-11-30

**Authors:** Zhenyu Li, Xinfei Li, Shubo Fan, Kunlin Yang, Chang Meng, Shengwei Xiong, Silu Chen, Zhihua Li, Xuesong Li

**Affiliations:** 1 Department of Urology Peking University First Hospital China Department of Urology, Peking University First Hospital, Institute of Urology, Peking University, National Urological Cancer Center, Beijing, China; Institute of Urology Peking University National Urological Cancer Center Beijing China

## Abstract

**Purpose:**

Horseshoe kidney (HSK) is the most common renal fusion anomaly, occurring in 0.25% of the population (1). It presents technical obstacles to pyeloplasty for ureteropelvic junction obstruction (UPJO) despite robotic assistance (2, 3). KangDuo-Surgical-Robot-01 (KD-SR-01), an emerging robotic platform in China, has yielded satisfactory outcomes in pyeloplasty (4, 5). We first describe our modified technique of robotic bilateral pyeloplasty for UPJO in HSK using KD-SR-01 system in the Lithotomy Trendelenburg position.

**Materials and Methods:**

A 36-year-old man with HSK and bilateral UPJO suffered right flank pain due to renal calculi (Figure-1). Repeated double-J stent insertion and ureteroscopy lithotripsy did not relieve his symptoms. A robot-assisted modified bilateral dismembered V-shaped flap pyeloplasty was performed using KD-SR-01 system in the Lithotomy Trendelenburg position.

**Results:**

Total operative time was 298 minutes with 50 ml estimated blood loss. There was no conversion to laparoscopic or open surgery. A follow-up of 14 months showed relieving symptoms and stable renal function. Cine magnetic resonance urography and computed tomography urography revealed improved hydronephrosis and good drainage. No intraoperative or postoperative complications occurred.

**Conclusions:**

It is technically feasible to perform a KD-SR-01-assisted modified bilateral dismembered V-shaped flap pyeloplasty in the Lithotomy Trendelenburg position for HSK. This procedure achieves managing UPJO on both sides without redocking the system and provides a wider operative field. In addition, it may be associated with better ergonomics, better cosmetic outcomes, and less possibility of postoperative bowel adhesion. However, further investigation is still warranted to confirm its safety, efficacy, and advantages over traditional procedures.

**Figure 1:**
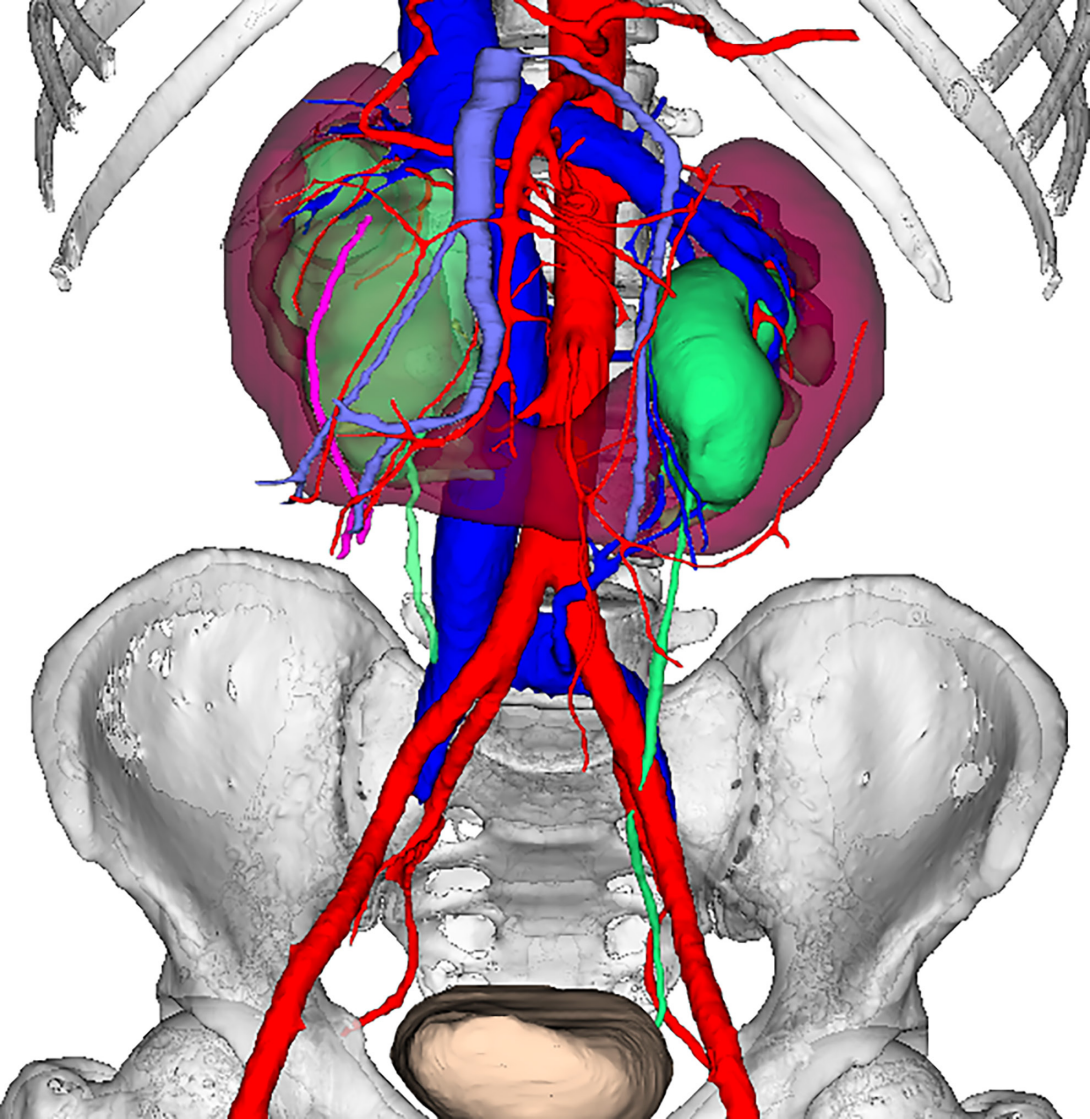
The three-dimensional image reconstructed by urinary enhanced computed tomography of the patient.
